# Carotid intima-media thickness, cardiovascular disease, and risk factors in 29,000 UK Biobank adults

**DOI:** 10.1016/j.ajpc.2025.101011

**Published:** 2025-05-19

**Authors:** Sayan Mitra, Raaj Kishore Biswas, Petra Hooijenga, Sophie Cassidy, Andrea Nova, Isabella De Ciutiis, Tian Wang, Cynthia M Kroeger, Emmanuel Stamatakis, Andrius Masedunskas, Raffaele De Caterina, Maria L Cagigas, Luigi Fontana

**Affiliations:** a Charles Perkins Centre, The University of Sydney, Sydney, NSW, 2006, Australia; b Central Clinical School, Faculty of Medicine and Health, The University of Sydney, Sydney, NSW, 2006, Australia; c CPC RPA Health for Life Program, Charles Perkins Centre, The University of Sydney, Sydney, NSW, 2006, Australia; d Mackenzie Wearables Research Hub, Charles Perkins Centre, The University of Sydney, Sydney, NSW, 2006, Australia; e School of Health Sciences, Faculty of Medicine and Health, The University of Sydney, Sydney, NSW, 2006, Australia; f Department of Brain and Behavioral Sciences, University of Pavia, 27100, Pavia, Italy; g Postgraduate School of Cardiology, Pisa University Hospital, University of Pisa; h Department of Endocrinology, Royal Prince Alfred Hospital, Sydney, NSW, 2050, Australia

**Keywords:** Atherosclerotic cardiovascular disease, Carotid intima-media thickness, Subclinical atherosclerosis, Major adverse cardiovascular events, Cardiometabolic risk factor, Coronary heart disease, Myocardial infarction, Heart failure, UK biobank, Prospective cohort study, Cardiometabolic-risk biomarker index, HbA1c, Total cholesterol ratio, Blood pressure, Lifestyle factors, Smoking, Physical activity, Cardiovascular disease prevention

## Abstract

**Importance:**

Atherosclerotic cardiovascular disease remains the leading cause of global morbidity and mortality. Identifying early markers of subclinical atherosclerosis is critical for predicting major adverse cardiovascular events (MACE) and improving prevention strategies. Carotid intima-media thickness (cIMT) is a well-established surrogate marker of atherosclerosis, but the impact of cardiometabolic risk factor burden on cIMT and future MACE risk is not fully understood.

**Objective:**

To assess the association between cIMT and the risk of MACE, and to evaluate the relationship between a composite cardiometabolic-risk biomarker index and cIMT as well as future MACE risk.

**Design:**

Prospective cohort study using data from the UK Biobank, with a median follow-up of 4.3 years.

**Setting:**

Population-based study of 29,292 participants from the UK Biobank.

**Participants:**

Men and women aged 40 to 69 years (n=29,292) free from cardiovascular disease at baseline. Exclusions were made for those with prior coronary heart disease, myocardial infarction, and heart failure.

**Exposures:**

Carotid intima-media thickness (cIMT) measured at baseline. A composite cardiometabolic-risk biomarker index (CRBI) was developed using HbA1c, total cholesterol ratio, and blood pressure.

**Main Outcomes and Measures:**

The primary outcomes were the risk of MACE, including coronary heart disease (CHD), myocardial infarction (MI), and heart failure (HF). The association between cIMT, CRBI, and the risk of these events was evaluated using hazard ratios (HRs) and adjusted for confounders.

**Results:**

Higher cIMT values (>800 µm) were predictive of increased risk for CHD (HR: 2.15 at 800 µm; 95 % CI: 1.07-4.31) and MI (HR: 2.46 at 800 µm; 95 % CI: 0.93-6.53). The cumulative burden of cardiometabolic risk factors, as measured by the CRBI score, was significantly associated with increased cIMT (β=44.38 µm for very high CRBI score; 95 % CI: 38.25-50.51; *p* < 0.001) and future MI risk (HR: 10.43 for very high CRBI score; 95 % CI: 3.18-34.24). Lifestyle factors such as smoking and physical activity were also correlated with higher cIMT, particularly in males.

**Conclusions:**

Carotid intima-media thickness is a strong predictor of coronary heart disease and myocardial infarction. The cumulative cardiometabolic-risk biomarker index offers additional predictive value for subclinical atherosclerosis and future cardiovascular events. These findings underscore the importance of comprehensive cardiometabolic health in CVD prevention strategies.

**Key Points**

Question: Is carotid intima-media thickness associated with major adverse cardiovascular events (MACE), and does a composite cardiometabolic-risk biomarker index improve prediction of future MACE risk?

Findings: In this prospective cohort study of 29,292 participants free from cardiovascular disease at baseline, higher cIMT values were associated with an increased risk of coronary heart disease and myocardial infarction. The composite cardiometabolic-risk biomarker index was significantly associated with increased cIMT and future myocardial infarction risk.

Meaning: Carotid intima-media thickness and a cumulative cardiometabolic-risk biomarker index are valuable predictors of cardiovascular events, highlighting their potential role in early risk assessment.

Alt-text: Unlabelled box

## 1 Introduction

Atherosclerotic cardiovascular disease (CVD) is the leading cause of morbidity, disability and mortality worldwide[1] In recent decades, a demographic and epidemiological transition has occurred not only in industrialized countries but also in developing nations adopting unhealthy Western diets and lifestyles, leading to a higher cumulative prevalence of atherosclerosis and its main complications, including myocardial infarction, ischemic stroke, heart failure, peripheral arterial disease and vascular dementia[2,3] Improving screening tools for the early detection of atherosclerotic CVD is crucial for predicting and preventing major adverse cardiovascular events (MACE). Currently, risk assessment in asymptomatic individuals can be performed using various CVD risk scores, such as the Framingham Risk Score,[4] as well as by non-invasively measuring the intima-media thickness of the common carotid artery (cIMT), a marker of subclinical atherosclerosis[5,6]

Accumulating evidence suggest that individual traditional CVD risk factors account for only a small proportion of the variance in cIMT, especially when measured in plaque-free locations[7] However, since major risk factors are additive in predictive power, assessing their cumulative burden may be more sensitive in capturing cIMT variance and the associated risk of developing MACE[8] Moreover, the impact of various lifestyle factors, including diet, physical activity, smoking, and sleep, on cIMT, independently of traditional cardiovascular risk factors, is not fully understood, particularly in individuals free of CVD[9] For example, studies have shown that adherence to a Mediterranean diet, is associated with reduced cIMT progression only among people with a high initial atherosclerotic burden[10] The UK Biobank Study, with its well-defined cohorts of carefully characterized middle-aged men and women, including high-quality cIMT measurements and extensive socio-demographic, lifestyle, and health metrics, provides a unique opportunity to examine factors that may modify cIMT and related cardiovascular risk.

In this study, we investigated the association between cIMT and atherosclerotic cardiovascular disease by risk factor burden among 14,720 men and 14,572 women from the UK Biobank free from CVD at baseline. First, we evaluated the prospective association between cIMT values and the risk of MACE. Second, we explored the association between a composite cardiometabolic-risk biomarker index comprising HbA1c, total cholesterol:HDL-cholesterol ratio (TC:HDLr), and blood pressure; and cIMT values. Finally, we assessed the prospective association between the composite cardiometabolic-risk biomarker index and the risk of MACE.

## 2 Methods

### 2.1 Study design and population

Details about the UK Biobank cohort have been previously published[11] Briefly, the UK Biobank cohort comprises 502,632 adults aged 40 to 69 who were assessed at 22 UK centers between March 2006 and December 2010, with a response rate of 5.5 %. Participants provided electronic consent and completed a touch-screen questionnaire covering socio-demographic, lifestyle, and health-related information. They also underwent a brief computer-assisted interview and provided anthropometric measurements and biological samples. Ethical approval was granted by the NHS Research Ethics Committee (Ref. 11/NW/0382) for UK Biobank research. The current analysis, conducted under project 62594, was approved by the UK Biobank research committee and adhered to STROBE reporting guidelines (**eTable 1** in Supplement).

### 2.2 Lifestyle factors and diet score

Full details of the study design and data extraction for each lifestyle factor collected at baseline (2006-10) are described in the Supplementary Materials (**eTable 2** in Supplement for definitions and **eFigure 5** in Supplement for the DAG). As previously described,[12] a continuous diet score was developed based on the consumption of individual food categories recorded in the food frequency questionnaire, with detailed information provided in **eTables 3-5** in Supplement[13,14] Alcohol consumption was derived by categorizing participants as “Never drinkers” or, for current drinkers, estimating the weekly units of alcohol intake by summing the consumption across different beverage types. Physical activity was categorized as “Low,” “Moderate,” or “High” based on the International Physical Activity Questionnaire (IPAQ) categorical score, which uses self-reported data on the number of days per week and the duration of walking, moderate, and vigorous activities[15] Sleep was classified into three groups: less than 7 hours per day, 7 to 9 hours per day (optimal), and more than 9 hours per day[16,17] Smoking status was categorized as “Current,” “Former,” or “Never” smokers. All biochemical markers were measured from blood samples collected at recruitment, and participants who regularly took cholesterol-lowering medications provided this information to an interviewer.

### 2.3 Cardiometabolic-risk biomarker index and score

We developed a simple integrated cardiometabolic-risk biomarker index (CRBI) as a proxy for optimal cardiometabolic health, incorporating three primary metabolic (non-behavioral) cardiovascular risk factors: HbA1c, TC:HDLr, and systolic (SBP) and diastolic (DBP) blood pressure collected at baseline (2006-10). These markers were selected for their well-known impact and widespread availability as screening tools for CVD[18] Following current international guidelines, HbA1c <5.7 % without antidiabetic medications was categorized as optimal, between 5.7 % and 6.4 % or <5.7 % but with antidiabetic medications as intermediate, and >6.4 % as poor[19] The TC:HDLr was considered optimal if <3.5 without taking lipid lowering medications, intermediate if between 3.5 and 5 or <3.5 with lipid lowering medications, and poor if >5[20] SBP was classified as optimal when <120 mmHg without taking antihypertensive medications, intermediate if between 120 and 139 mmHg or <120 mmHg with antihypertensive medications, and poor when >139 mmHg; DBP was optimal if <80 mmHg without taking antihypertensive medications, intermediate if between 80 and 89 mmHg or <80 mmHg with antihypertensive medications, and poor if ≥90 mmHg[21] Detailed definitions and categorizations of this index are outlined in **eTable 6**. For the HbA1c and TC:HDLr variables, a study participant could either score 0 (optimal), 1 (intermediate), or 2 (poor) points. Blood pressure readings were evaluated separately: SBP and DBP were each assigned 0 points for optimal, 0.5 points for intermediate, and 1 point for poor. Therefore, the CRBI score ranged from 0 (optimal) to 6 (poor). Finally, we stratified participants a priori into mutually exclusive categories based on whether they had all optimal factor levels (score 0), low (scores >0 and ≤1), moderate (scores >1 and ≤2), high (scores >2 and ≤3), and very high (score >3), as detailed in **eTable 7**. For instance, a participant with optimal scores for all biomarkers (HbA1c <5.7 %, TC:HDLr <3.5, and BP <120/80 mmHg) would have a CRBI score of 0, indicating optimal cardiometabolic score. This approach allows for a comprehensive assessment of an individual's overall cardiometabolic health by integrating continuously distributed biological biomarkers into a single index.

#### 2.3.1 Carotid intima-media thickness

Carotid intima-media thickness measurements, collected during imaging visits between 2014-2018, were measured using a CardioHealth Station (Panasonic Biomedical Sales Europe BV, Leicestershire, UK) with participants lying down with their heads elevated at a 45° angle. Automated cIMT measurements were obtained from 2-dimensional carotid scans on both the transverse (short axis) and longitudinal (long axis) planes. Four cIMT readings were taken at specific angles: 120° and 150° for the right carotid artery, and 210° and 240° for the left carotid artery. For each angle, the minimum, mean, and maximum values were recorded[22] The cIMT values for the four angles were averaged, considering the minimum, mean, and maximum values for our analysis. Quality control of cIMT measurements was performed by the UK Biobank, with validation both internally and externally using predefined criteria[22,23] cIMT measurements that failed quality control, either due to values of zero or flagged inconsistencies, were excluded from the analysis[24] At the time of our study, cIMT measurements were available for 49,112 participants who had completed the second follow-up visit for the UK Biobank. We included participants with available cIMT data and covariate information from the first follow-up (*n* = 29,292)[25]

#### 2.3.2 Cause-specific incidence and CVD mortality

Cardiovascular diagnoses were derived from linked Hospital Episode Statistics using the International Classification of Diseases, 10th Revision (ICD-10) code ‘G20’ (**eTable 8** in Supplement). Participants were linked to mortality registries through the UK National Health Service Central Registry, and underlying causes of death were extracted from death certificate data, coded according to ICD-10. The outcomes examined in this study included coronary heart disease (CHD) (ICD-10 code I25), myocardial infarction (MI) (I21, I22, I23), heart failure (HF) (I50), aortic aneurysm (I71), peripheral vascular disease (I73), stroke (I63), and all-cause dementia (A81.0, F00, F01, F02, F03, F05, G30, G31.0, G31.1, G31.8, and I67.3). Participants with records of these conditions at baseline were excluded from the analysis for that specific outcome. Participants were followed up through 30 November 2022.

### 2.4 Statistical analysis

Cohort characteristics were summarized using mean (standard deviation [SD]) or median (interquartile range [IQR]) for continuous variables, and percentages for categorical variables. The associations between time-to-event dose-response of cIMT and seven disease outcomes, including both incidence and mortality, were examined through a complete case analysis. Hazard ratios (HRs) were calculated using Fine-Gray sub-distribution models to account for competing risks from non-disease-specific deaths[26] Due to the skewed distribution of primary exposures, knots were placed at equally distributed frequencies (10^th^, 33^rd^, and 67^th^ percentiles) in areas of higher data density[27] Departure from linearity was assessed by a Wald test. Proportional hazard assumptions were tested using Schoenfeld Residuals, with no violations observed (all *p* > 0.05).

Core models adjusted for age, sex, ethnicity, C-reactive protein, Townsend deprivation index, sleep duration, physical activity (IPAQ-derived), diet, cIMT, smoking, alcohol consumption, and body weight, with both adjusted and unadjusted results reported. The model was repeated with additional adjustment for the CRBI score. Linear regression assessed the association between cIMT and CRBI score, controlling for lifestyle factors and confounders. Multicollinearity was tested using variance inflation factors (VIF), and β coefficients, 95 % confidence intervals, and p-values were reported. Sex differences in lifestyle factor effects were explored.

To evaluate the robustness of the association between physical activity and cIMT, we conducted sensitivity analyses by adjusting for different subsets of covariates, including BMI, blood pressure, and lipid profile. Additionally, to assess potential reverse causation, we tested models that excluded participants with high cardiovascular risk at baseline.

In our primary models, we adjusted for body weight rather than BMI, as total body weight may more accurately reflect overall metabolic load and cardiovascular stress, independent of height. However, recognizing that BMI is a widely used cardiovascular risk marker, we performed a sensitivity analysis in which body weight was replaced with BMI across all models.

In all models, the reference points for CRBI score and cIMT were optimal (0) and the lowest value (476.8 µm), respectively. The model was also applied to individual CRBI components (blood pressure, TC:HDLr, HbA1c). cIMT values were truncated at the 99^th^ percentile, and participants with events in the first year or prevalent diseases at baseline were excluded to avoid reverse causation. Detailed analyses and results are available in the Supplement. All analyses were performed using R (version 4.4.0).

## 3 Results

### 3.1 Baseline characteristics

The analysis included a total of 29,292 participants after excluding those with a history of CHD (*n* = 1483), MI (*n* = 745), and HF (*n* = 285) at baseline and all study covariates were available (**eFigure 1** in Supplement). The mean age was 64±7.8 years, 48 % were women, and 93.8 % reported White ethnicity (**eTable 9** in Supplement). Participants with higher cIMT were older and had higher BMI, waist circumference, total cholesterol, LDL-cholesterol, triglycerides, blood pressure, HbA1c, C-reactive protein, and use of antihypertensive medication and statins **(eTable 9** in Supplement**).**

### 3.2 Carotid IMT as a predictor of major adverse cardiovascular events

**Fig. 1:**
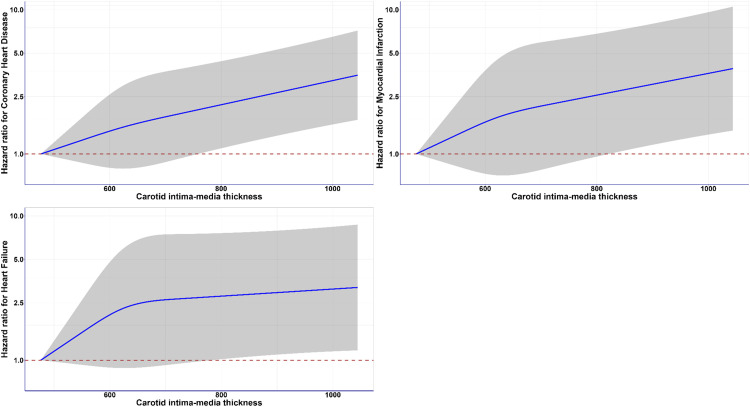
Association of carotid intima media thickness and CVD incidence (coronary heart disease, myocardial infarction, and heart failure), (N=29292).

**Legend:** Analyses were adjusted for age, sex, ethnicity, C-reactive protein, Townsend deprivation index, sleep duration, physical activity (derived from IPAQ), dietary consumption, cardiometabolic risk biomarker index (CRBI) score, smoking, alcohol consumption, and body weight. All analyses excluded participants who had an event in the first year of follow-up and those with prevalent outcome-specific diseases at the cIMT baseline were excluded. Reference for cIMT was set at minimum (476.8 µm). The shaded region indicates the 95 % confidence interval (CI). Within this shaded area, the solid line in blue represents the hazard ratio (HR).

We observed cIMT as a predictor for coronary heart disease, myocardial infarction, and heart failure (Table 1; Fig. 1), but not for stroke, dementia, peripheral vascular disease, and aortic aneurysm (**eFigure 2** in Supplement). During a median follow-up of 4.3 years, there were 345, 203 and 232 new cases of CHD, MI, and HF, respectively. We observed a near-linear dose response association between cIMT and event of CHD and MI in the adjusted dose response plots (Fig. 1). The hazard ratio (HR) for CHD was twofold (HR: 2.15, 95 % CI: 1.07, 4.31) at 800 µm and threefold (HR: 3.15, 95 % CI: 1.57, 6.35) at 1000 µm. HR for myocardial infarction (MI) was 2.46 (95 % CI: 0.93, 6.53) at 800 µm, and 3.46 (95 % CI: 1.31, 9.15) at 1000 µm. A similar pattern was observed for heart failure (HF) (HR: 2.74 at 800 µm and HR: 3.06 at 1000 µm) (Table 1).

**Table 1:** Point estimates of dose-response association between cIMT and MACE, where the cIMT reference point is 476.8 µm.

cIMT (µm)	Hazard ratios (95 % CI)		
	CHD	MI	HF
**477** (reference)	1.00 (1.00, 1.00)	1.00 (1.00, 1.00)	1.00 (1.00, 1.00)
**500**	1.07 (0.95, 1.20)	1.10 (0.93, 1.29)	1.15 (0.97, 1.36)
**600**	1.42 (0.79, 2.55)	1.62 (0.71, 3.68)	2.04 (0.88, 4.75)
**700**	1.78 (0.86, 3.66)	2.07 (0.75, 5.72)	2.59 (0.91, 7.35)
**800**	2.15 (1.07, 4.31)	2.46 (0.93, 6.53)	2.74 (1.00, 7.50)
**900**	2.61 (1.31, 5.18)	2.92 (1.11, 7.63)	2.89 (1.07, 7.78)
**1000**	3.15 (1.57, 6.35)	3.46 (1.31, 9.15)	3.06 (1.13, 8.27)

CHD, coronary heart disease; MI, myocardial infarction; HF, heart failure.

Analyses adjusted for age, sex, ethnicity, C-reactive protein, Townsend deprivation index, sleep duration, physical activity derived from IPAQ, dietary consumption, composite biomarker score (HbA1c, total cholesterol to HDL ratio, and blood pressure), smoking, alcohol consumption, and body weight.

### 3.3 Cumulative burden of cardiometabolic risk factors as predictor of cIMT

**Fig. 2:**
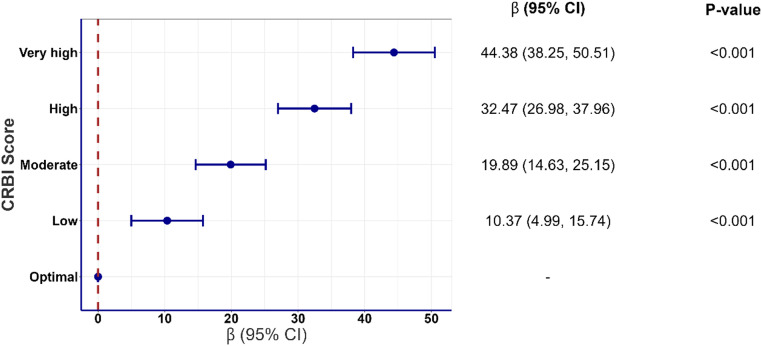
Association between cardiometabolic risk biomarker index (CRBI) score and carotid intima-media thickness (cIMT), (N=29292).

**Legend:** CRBI score, cardiometabolic risk biomarker index score. Data are presented as beta coefficients with 95 % confidence intervals. CRBI scores of 0, ≥0 & ≤1, >1 & ≤2, >2 & ≤3, >3 correspond to optimal, low, moderate, high, and very high. Analyses adjusted for age, sex, ethnicity, C-reactive protein, Townsend deprivation index, sleep duration, physical activity derived from IPAQ, dietary consumption, smoking, alcohol consumption, and body weight. We removed participants with a previous history of CHD, MI, or HF. All analyses excluded participants who had an event in the first year of follow-up.

The cumulative burden of cardiometabolic risk factors, as assessed by the CRBI score, was strongly associated with increased cIMT (Fig. 2). Stratifying participants by their CRBI scores revealed a sharp rise in cIMT from those with optimal risk factor levels to those with very high CRBI scores (*p* < 0.001). Compared to individuals with optimal cardiometabolic health representing 2 % of men and 14 % of women in our sample (**eFigure 3** in Supplement), those classified as having a 'very high' CRBI score (12 % of men and 5 % of women) showed a higher cIMT (β=44.38 µm; 95 % CI: 38.25 to 50.51; *p* < 0.001). The correlation between cIMT and the CRBI score was more pronounced than with any individual biomarker component (**eTable 11** in Supplement).

### 3.4 CRBI score as a predictor of future MACE

**Fig. 3:**
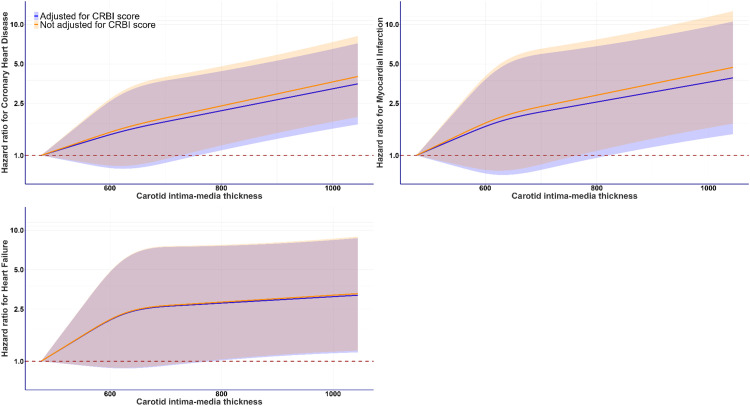
Adjustment for the CRBI score attenuated the magnitude of the associations between cIMT and CVD event risk, (N=29292).

**Legend:** Analyses were adjusted for age, sex, ethnicity, C-reactive protein, Townsend deprivation index, sleep duration, physical activity (derived from IPAQ), dietary consumption, smoking, alcohol consumption, and body weight. All analyses excluded participants who had an event in the first year of follow-up and those with prevalent outcome-specific diseases at the cIMT baseline were excluded. Reference for cIMT was set at minimum (476.8 µm). The shaded region indicates the 95 % confidence interval (CI). Within this shaded area, the solid lines in blue and orange represents the hazard ratio (HR).

The CRBI score, reflecting the cumulative burden of cardiometabolic risk factors, predicted a higher likelihood of developing CHD and MI, but not HF (Table 2). The risk of MI rose linearly from individuals with optimal score factor levels to those with a very high CRBI score. Compared to those with optimal cardiometabolic health, individuals categorized as having a 'very high’ CRBI score' had a sharp increase in MI risk (HR: 10.43, 95 % CI: 3.18 to 34.24) after adjusting for multiple confounders. A similar but comparatively less pronounced effect was observed for CHD (HR: 2.70, 95 % CI: 1.49 to 4.91). Adjustment for CRBI score did not greatly attenuate the magnitude of the associations between cIMT and CVD event risk, as well as their statistical significance (Fig. 3).

**Table 2:** Point estimates of dose-response association between CRBI score and MACE, where CRBI score reference point is optimal (HbA1c <5.7 %, TC:HDLr <3.5, BP <120/80).

CRBI score	Hazard ratios (95 % CI)		
	CHD (Event=345)	MI (Event=203)	HF (Event=232)
**Optimal** (reference)	1.00 (1.00, 1.00)	1.00 (1.00, 1.00)	1.00 (1.00, 1.00)
**Low**	1.33 (0.90, 1.97)	2.98 (1.37, 6.50)	1.00 (0.63, 1.58)
**Moderate**	1.72 (0.93, 3.20)	6.50 (1.86, 22.64)	1.02 (0.50, 2.11)
**High**	2.16 (1.20, 3.92)	8.33 (2.48, 28.04)	1.10 (0.56, 2.20)
**Very high**	2.70 (1.49, 4.91)	10.43 (3.18, 34.24)	1.19 (0.60, 2.39)

CRBI score, cardiometabolic risk biomarker index score.

CRBI scores of 0, ≥0 & ≤1, >1 & ≤2, >2 & ≤3, >3 correspond to optimal, low, moderate, high, and very high.

CHD, coronary heart disease; MI, myocardial infarction; HF, heart failure.

Analyses adjusted for age, sex, ethnicity, C-reactive protein, Townsend deprivation index, sleep duration, physical activity derived from IPAQ, dietary consumption, cIMT, smoking, alcohol consumption, and body weight.

### 3.5 Associations between lifestyle factors and cIMT

Risk estimates of cIMT for lifestyle factors are summarized in **eTable 12** in Supplement. After adjusting for multiple confounding factors, smoking was associated with higher cIMT, with both former (β=10.16 µm; 95 % CI: 7.44 to 12.89) and current smokers (β=14.71 µm; 95 % CI: 7.74 to 21.70) having a higher cIMT compared to those who never smoked. Additionally, the analyses revealed a non-linear detrimental association of self-reported physical activity (measured by IPAQ). Compared to participants with low levels of physical activity, those who engaged in high physical activity had a 12.01 µm (95 % CI: 8.43 to 15.59) higher cIMT. Sex-based stratification, shown in **eTable 13** in Supplement, showed that the detrimental effect of physical activity was present in males but not in females. Furthermore, sleep and diet quality were not correlated with changes in cIMT. During a median follow-up of 4.3 years, there were 19 cases of ischemic stroke recorded. Although the HR for stroke at higher cIMT values suggested an increased risk (HR ∼2 at 800 µm, **eFigure 2** in Supplement), the confidence intervals were wide, and the association did not reach statistical significance.

### 3.6 Sensitivity analyses

Our sensitivity analyses confirmed that the association between physical activity and cIMT remained statistically significant across different covariate models, with only slight variations in effect size. Replacing body weight with BMI yielded similar results, with no significant changes in the observed associations between cIMT and cardiovascular risk factors. These findings suggest that the relationships identified are robust to different measures of adiposity.

## 4 Discussion

This large prospective study of over 29,000 participants free of cardiovascular disease at baseline provides a comprehensive analysis of the relationship between carotid intima-media thickness (cIMT), cardiometabolic risk factors, and major adverse cardiovascular events. Higher cIMT was linked to an increased risk of coronary heart disease (CHD), myocardial infarction (MI), and heart failure, but not stroke, dementia, peripheral vascular disease, or aortic aneurysm. The near-linear dose-response for CHD and MI highlights cIMT's value as a predictive marker, particularly when combined with the cumulative cardiometabolic risk biomarker index (CRBI), which was a stronger predictor than individual risk factors. These findings underscore the importance of optimizing cardiometabolic health to reduce cIMT and cardiovascular risk.

The most striking findings in this analysis are the substantial differences in atherosclerosis, as assessed by cIMT, and cardiovascular risk between participants with optimal risk factor levels and those with major abnormal risk factors. The CRBI score's strong association with cIMT and cardiovascular events echoes the findings of studies that emphasize the importance of cumulative risk assessment over individual risk factors. The growing emphasis on maintaining optimal or very low levels of traditional risk factors to reduce CVD morbidity and mortality, and to extend healthspan and lifespan, highlights the impact of favorable cumulative risk profiles on cardiovascular biology and the reduced risk of developing CHD and MI[28] Moderate calorie restriction, with adequate intake of vitamins and minerals, markedly improves multiple key cardiometabolic risk factors well below conventional risk thresholds used in clinical practice, with endurance exercise training providing additional independent benefits for HDL-cholesterol and glucose metabolism[[29], [30], [31]]

Our findings align with previous research identifying cIMT as a predictor of cardiovascular events,[32,33] although we could not observe an association between cIMT and stroke likely due to the low event number. The Rotterdam Elderly Study, with 3,996 participants and a 6.1-year follow-up, identified cIMT as a significant independent predictor for both CHD and stroke[34] Similarly, a study involving 4,476 subjects 65 years of age or older over a median follow-up period of 6.2 years demonstrated that both common and internal cIMT measurements predicted CHD and stroke[35] More studies are needed to confirm these associations and further elucidate the mechanisms underlying the relationship between cIMT and cardiovascular events, particularly stroke. Indeed, accumulating data suggest that increased IMT in the common and internal carotid arteries might reflect different underlying pathophysiological mechanisms and therefore be differently associated with CHD and stroke risk[36]

In examining lifestyle factors, our analysis suggests that smoking, rather than diet quality, alcohol intake, or sleep, is likely associated with increased cIMT. Both former and current smokers tended to have higher cIMT levels compared to those who had never smoked, aligning with previous studies indicating a negative vascular impact of smoking[32,37] Notably, dietary intake was measured using a food frequency questionnaire by food groups, which did not consider macronutrient ratios (carbohydrate, protein, and fat), types (saturated, unsaturated, etc.), and energy intake. Although we did not find an association between dietary intake and cIMT, there was a notable association between worsening plasma lipids and increasing cIMT.

Our study uncovered a nonlinear relationship between physical activity and cIMT, with higher activity levels associated with increased cIMT, particularly among males. This aligns with recent observations that lifelong endurance athletes may have more coronary plaques, including non-calcified ones[[38], [39], [40]] Preclinical data suggest that long-term intensive training, unlike moderate exercise, may adversely affect arterial structural and function through activation of the renin-angiotensin-aldosterone system[41] In some individuals, overactivation of the sympathetic and angiotensin systems could contribute to abnormal plaque remodeling at higher levels of physical activity. The positive association observed between physical activity and cIMT was unexpected, as previous studies, particularly randomized controlled trials, have generally reported an inverse relationship. However, most of these studies focused on structured exercise interventions, whereas our analysis examined self-reported habitual physical activity. One possible explanation is reverse causation, where individuals with higher cardiovascular risk may engage in more physical activity following medical advice. Additionally, some residual confounding cannot be ruled out, as suggested by our sensitivity analyses. Future research using device-measured physical activity to capture detailed activity types and intensities, along with longitudinal study designs, is needed to better understand this relationship.

This study’s strengths include its large sample size, thorough assessment of cardiometabolic risk factors and cIMT, and the exclusion of individuals with early cardiovascular events to mitigate reverse causation. However, certain limitations should be considered when interpreting the findings. As an observational study, causal inferences cannot be drawn. The self-reported lifestyle data varied in quality, and baseline-only measurements did not account for changes over time. While the CRBI score integrates multiple well-established cardiometabolic risk factors, like all novel risk indices, it involves some degree of subjectivity in its construction. Therefore, further validation in independent cohorts and external datasets is required to confirm its predictive value across different populations before its clinical applicability can be fully established. The lack of a statistically significant association between cIMT and stroke is likely due to the relatively low number of recorded stroke events, leading to reduced statistical power and wider confidence intervals. Additionally, stroke includes different subtypes, such as ischemic and hemorrhagic, which may have distinct pathophysiological mechanisms not fully captured by cIMT. Larger studies with longer follow-up periods, such as Baldassarre et al.,[42] have reported more robust associations. Future research with extended follow-up and detailed stroke subtype classification may help clarify the role of cIMT in stroke risk prediction. Although follow-up cIMT data were available from the UK Biobank's First Repeat Imaging Visit (beginning in 2019, with approximately 5,400 participants), we used data only from the initial Imaging Visit (starting in 2014, with approximately 52,000 participants) due to the limited sample size and insufficient follow-up duration to reliably model repeated-measures survival analyses. Additionally, the study population was predominantly white and drawn from a single country, which may limit generalizability and leave certain environmental, genetic, and cultural confounders unaccounted for.

In summary, our study of ∼30,000 cardiovascular disease-free participants shows that cIMT is associated with major adverse cardiovascular events, especially CHD, MI, and HF. Higher cIMT levels correlate strongly with cumulative cardiometabolic risk, highlighting the value of comprehensive risk factor assessment via the CRBI score in predicting outcomes. Routine cIMT evaluation, particularly in high-risk individuals, may enhance risk stratification beyond traditional scores, aiding earlier intervention in subclinical atherosclerosis. While recognizing the study’s observational nature and potential confounders, these findings underscore importance of managing multiple risk factors through lifestyle and pharmacological strategies. Future research in diverse populations is needed to refine prevention and risk prediction.

**Figure:**
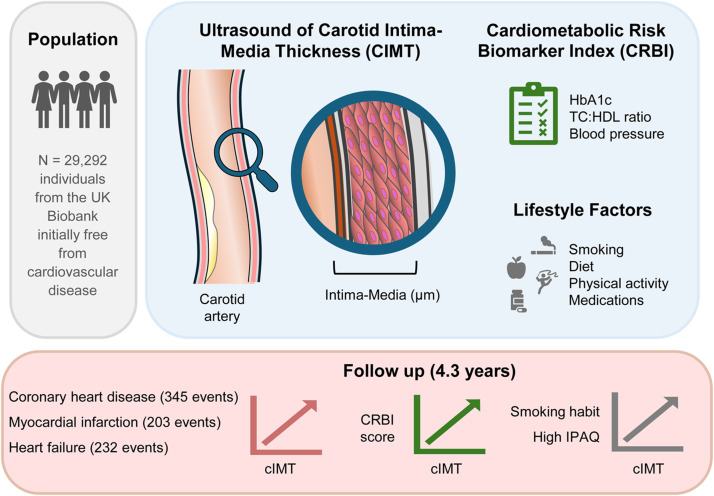
Central illustration: Carotid intima-media thickness (cIMT), measured in 29,292 UK Biobank participants free of cardiovascular disease, was examined against a composite cardiometabolic risk index (HbA1c, blood pressure, TC:HDL ratio), lifestyle factors, and cardiovascular outcomes. Higher cardiometabolic risk and smoking were linked to greater cIMT, while high physical activity showed a modest positive association. Over 4.3 years, elevated cIMT independently predicted coronary heart disease, myocardial infarction, and heart failure.

## CRediT authorship contribution statement

**Sayan Mitra:** Writing – review & editing, Writing – original draft, Visualization, Validation, Software, Resources, Project administration, Methodology, Investigation, Formal analysis, Data curation, Conceptualization. **Raaj Kishore Biswas:** Writing – review & editing, Validation, Supervision, Methodology, Formal analysis, Data curation. **Petra Hooijenga:** Methodology. **Sophie Cassidy:** Writing – review & editing. **Andrea Nova:** Methodology, Data curation. **Isabella De Ciutiis:** Writing – review & editing. **Tian Wang:** Writing – review & editing. **Cynthia M Kroeger:** Writing – review & editing. **Emmanuel Stamatakis:** Writing – review & editing. **Andrius Masedunskas:** Writing – review & editing. **Raffaele De Caterina:** Writing – review & editing. **Maria L Cagigas:** Writing – review & editing. **Luigi Fontana:** Writing – review & editing, Validation, Supervision, Resources, Methodology, Conceptualization.

## Declaration of competing interest

The authors declare that they have no known competing financial interests or personal relationships that could have appeared to influence the work reported in this paper.

## Appendix Supplementary materials

Image, application 1
