# Supplementary material for: Carotid intima-media thickness, cardiovascular disease, and risk factors in 29,000 UK Biobank adults
Source: Am J Prev Cardiol. 2025 May 19;22:101011. doi: 10.1016/j.ajpc.2025.101011 (PMC12162042; doi:10.1016/j.ajpc.2025.101011)
Supplement: Supplementary file 1 [file mmc1.pdf]

Supplementary materials

Carotid intima-media thickness, cardiovascular disease, and risk factors in 29,000 UK Biobank adults

Table of Contents

eFigure 1 Flow diagram of participants.

eFigure 2 Associations of CIMT and stroke (n=19), dementia (N=78), peripheral vascular disease (N=110), and aortic aneurysm (N=74) after adjusting for CRBI.

eFigure 3 Distribution of cardiometabolic-risk biomarker index score by sex.

eFigure 4 Carotid intima-media thickness values per CRBI score in our study population.

eFigure 5 Directed acyclic graph (DAG) showing variables for the cardiometabolic-risk biomarker index (CRBI) from UK Biobank biomarkers, and its association with vascular ageing via carotid intima-media thickness (CIMT).

eTable 1 STROBE statement.

eTable 2 Covariates, units, type, definitions, normal values, and UK Biobank field codes (if applicable).

eTable 3 Food groups (used for diet score creation).

eTable 4 Diet score calculations based on food groups from FFQ.

eTable 5 Diet groups based on dietary consumption.

eTable 6 Cardiometabolic-risk biomarker index (CRBI).

eTable 7 Cardiometabolic-risk biomarker index (CRBI) score.

eTable 8 ICD-10 codes for outcomes and UK Biobank data fields.

eTable 9 Participant descriptive characteristics by cIMT quartiles, (N=29292).

eTable 10 Multicollinearity for linear regression between CMIT and all factors.

eTable 11 Association of CRBI score and individual biomarkers with cIMT.

eTable 12 Association between cIMT and covariates, (N=29292).

eTable 13 Results from the adjusted model stratified by sex. (N=29,292).

Supplementary references

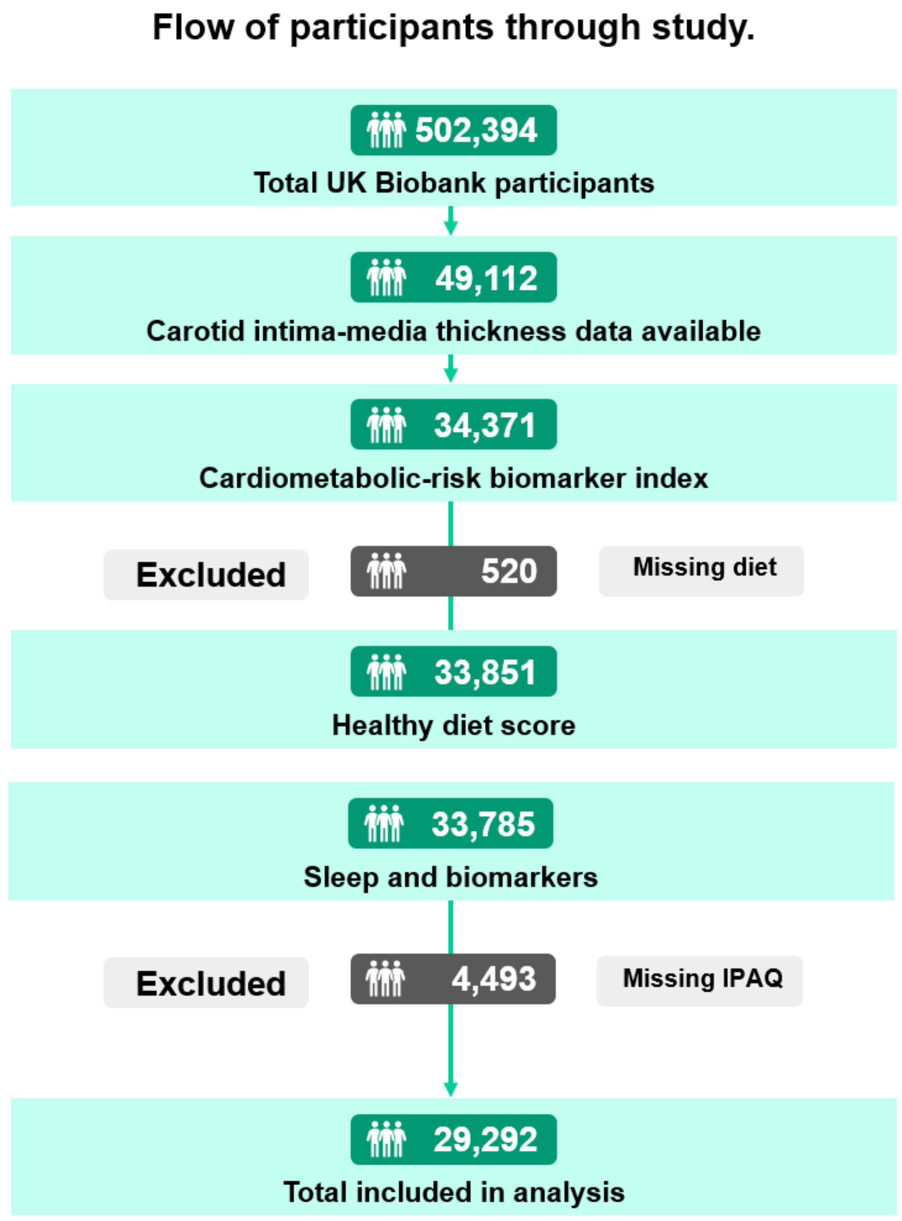

eFigure 1 Flow diagram of participants.

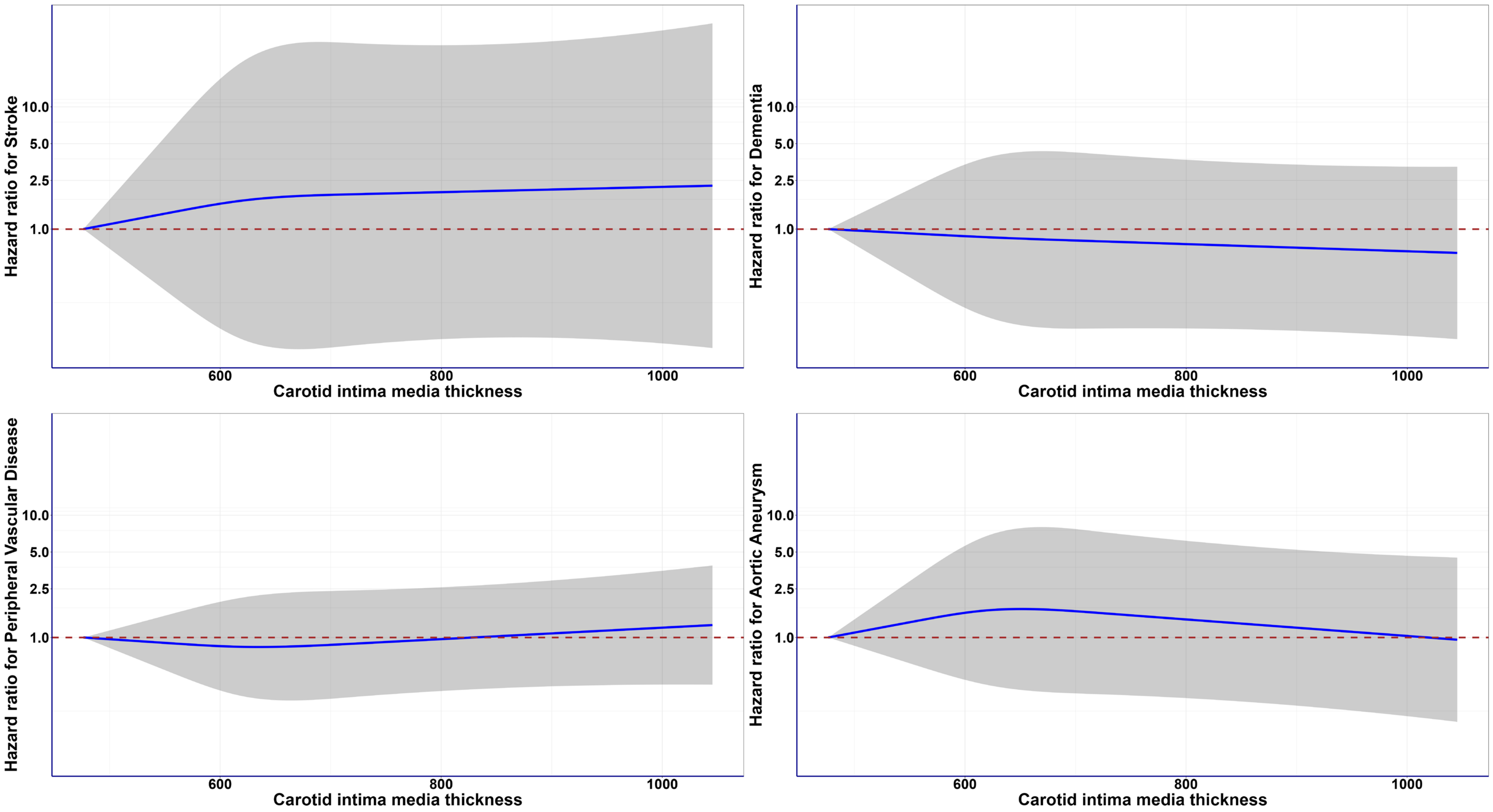

eFigure 2 Associations of CIMT and stroke (n=19), dementia (N=78), peripheral vascular disease (N=110), and aortic aneurysm (N=74) after adjusting for CRBI.

Supplementary

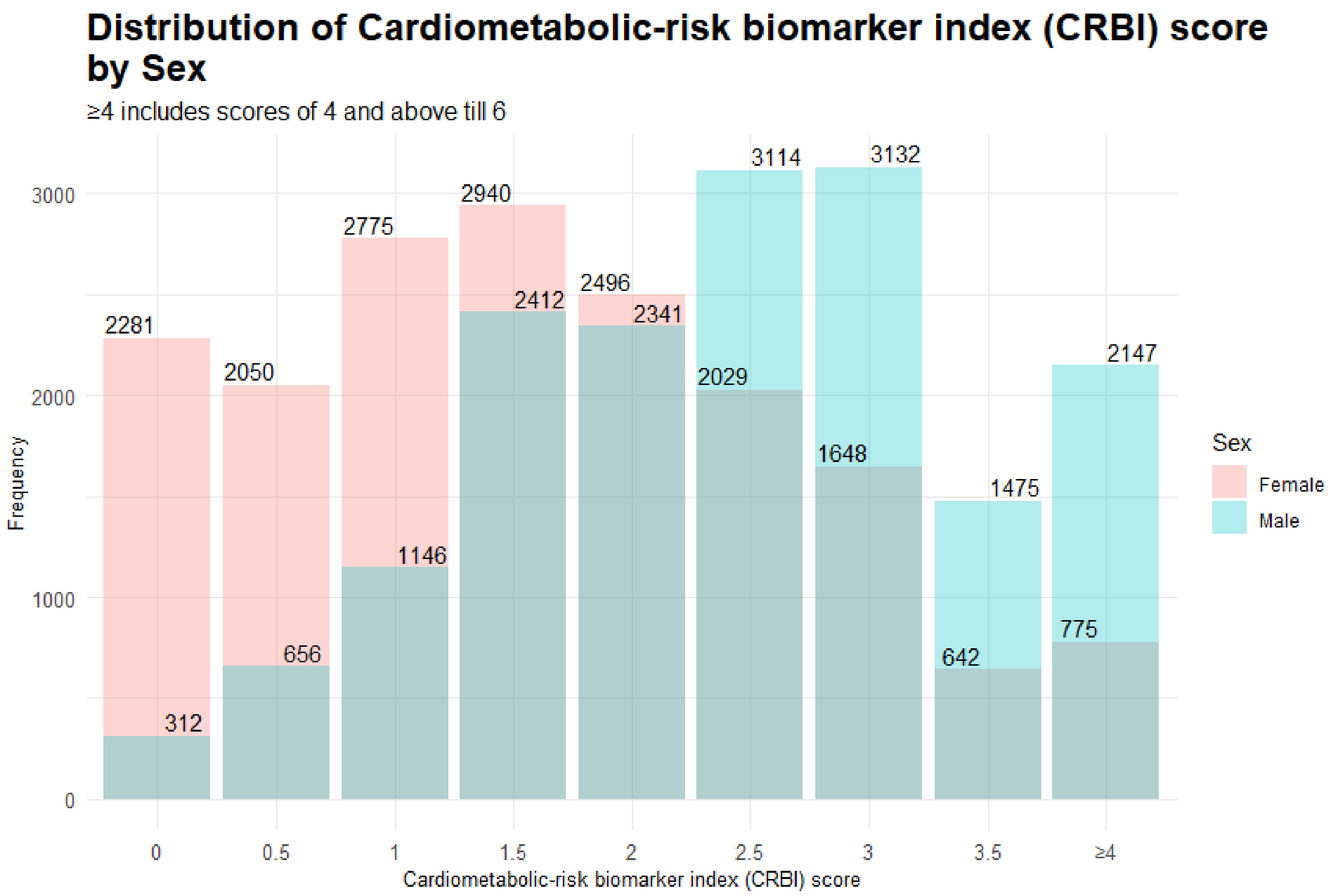

eFigure 3 Distribution of cardiometabolic-risk biomarker index score by sex.
CRBI scores of 0, >0 & ≤1, >1 & ≤2, >2 & ≤3, >3 correspond to optimal, low, moderate, high, and very high respectively.

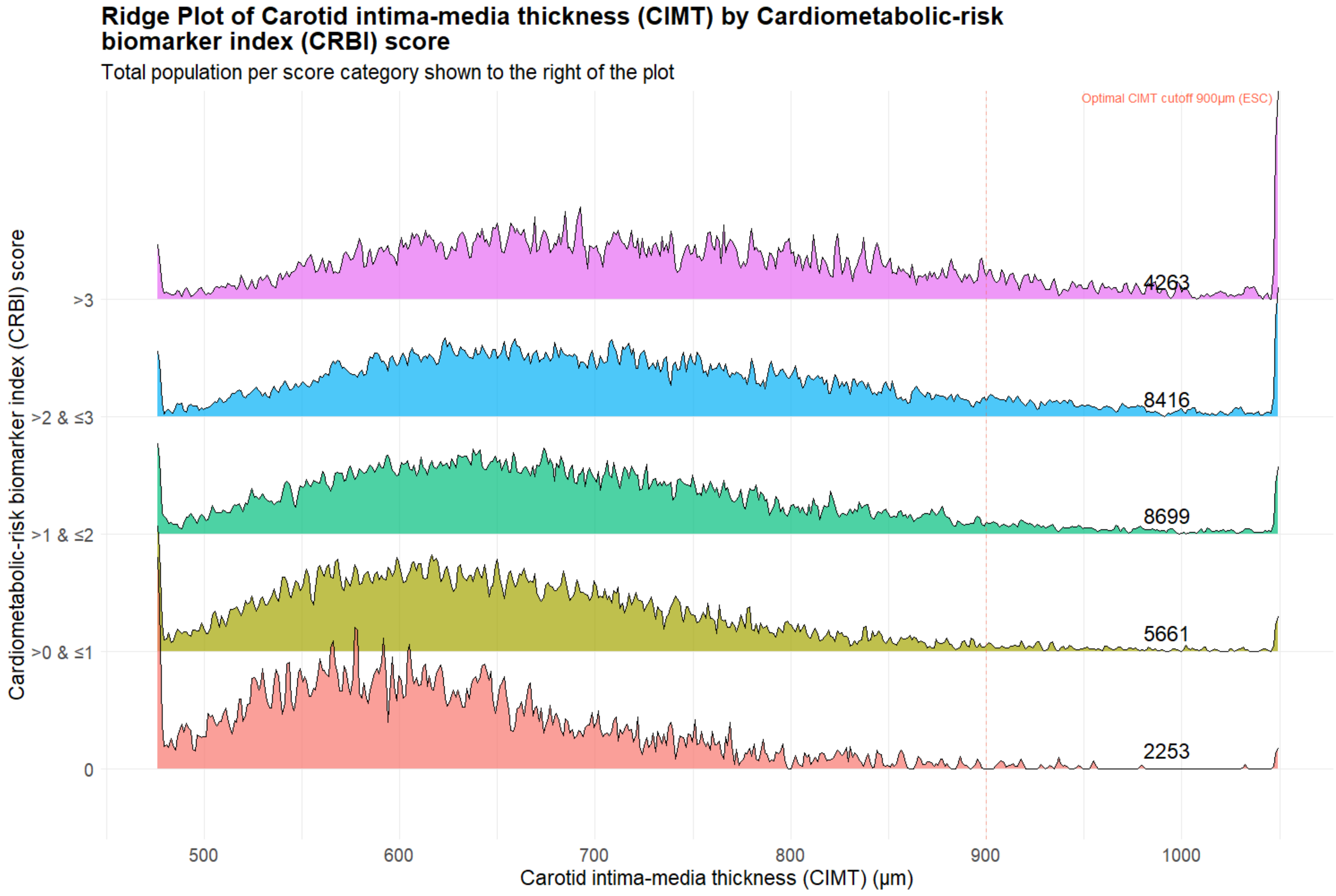

eFigure 4 Carotid intima-media thickness values per CRBI score in our study population.

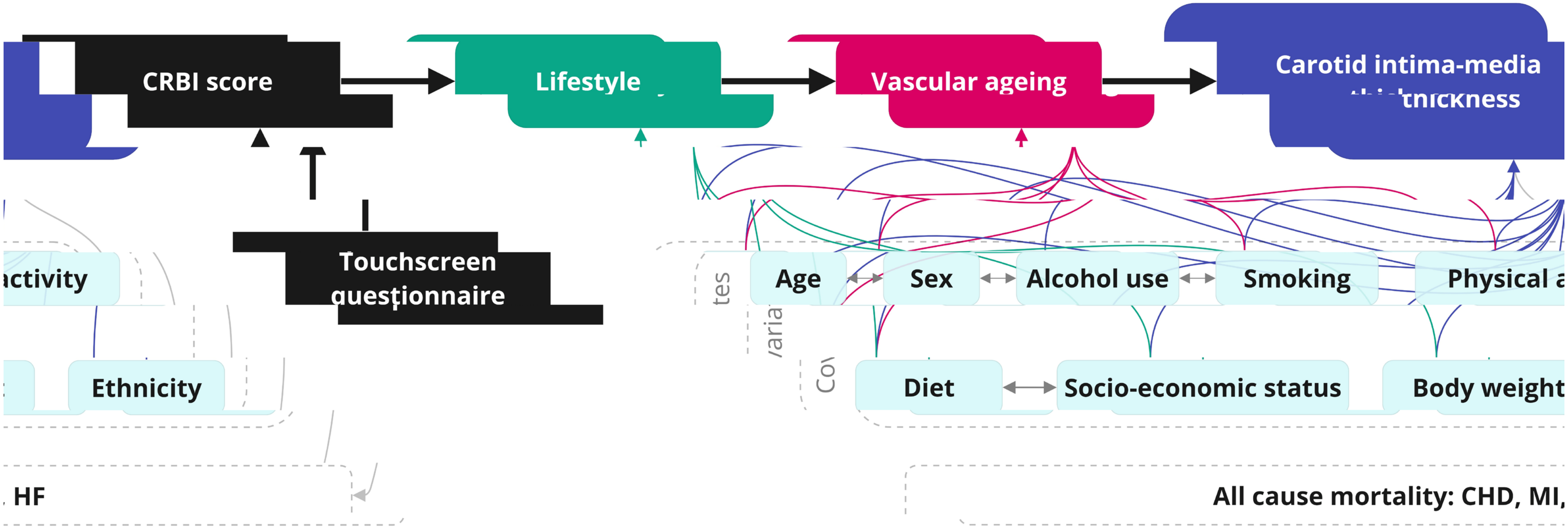

eFigure 5 Directed acyclic graph (DAG) showing variables for the cardiometabolic-risk biomarker index (CRBI) from UK Biobank biomarkers, and its association with vascular ageing via carotid intima-media thickness (CIMT).

In this Directed acyclic graph (DAG), the Cardiometabolic-risk biomarker index (CRBI) score is calculated based on biomarker values from the UK Biobank. The CRBI score is used to measure health status, which is then associated with vascular ageing. Vascular ageing is assessed by carotid intima-media thickness (CIMT).

Other covariates such as age, sex, diet, socio-economic status, physical activity, smoking, alcohol use, and body weight are also included as additional nodes that may have direct or indirect effects on vascular ageing. Outcomes and exposures are in differently colored bubbles.

CHD, coronary heart disease; MI, myocardial infarction; HF, heart failure

Supplementary

eTable 1 STROBE statement.

STROBE Statement—Checklist of items that should be included in reports of ***cohort studies***

	Item No	Recommendation	Location in manuscript
**Title and abstract**	1	(*a*) Indicate the study's design with a commonly used term in the title or the abstract	Page 1 Line 1, and Page 3 Line 48
		(*b*) Provide in the abstract an informative and balanced summary of what was done and what was found	Page 3 Lines 45 to 66
**Introduction**			
Background/rationale	2	Explain the scientific background and rationale for the investigation being reported	Page 4 Lines 72 to 93
Objectives	3	State specific objectives, including any prespecified hypotheses	Page 4 Lines 94 to 100
**Methods**			
Study design	4	Present key elements of study design early in the paper	Page 4 Line 104 to Page 5 Line 112
Setting	5	Describe the setting, locations, and relevant dates, including periods of recruitment, exposure, follow-up, and data collection	Page 4 Line 104 to Page 5 Line 112
Participants	6	*(a)* Give the eligibility criteria, and the sources and methods of selection of participants<br>*(b)* For matched studies, give matching criteria and number of exposed and unexposed	eFigure 1 in the Supplement
Variables	7	Clearly define all outcomes, exposures, predictors, potential confounders, and effect modifiers. Give diagnostic criteria, if applicable	eTable 2 in the Supplement
Data sources/ measurement	8*	For each variable of interest, give sources of data and details of methods of assessment (measurement). Describe comparability of assessment methods if there is more than one group	eTable 2 in the Supplement
Bias	9	Describe any efforts to address potential sources of bias	NA
Study size	10	Explain how the study size was arrived at	Page 6 Line 167-169
Quantitative variables	11	Explain how quantitative variables were handled in the analyses. If applicable, describe which groupings were chosen and why	Page 6 Line 172 to Page 6 Line 181

Statistical methods	12	(*a*) Describe all statistical methods, including those used to control for confounding	Page 6 Line 184
		(*b*) Describe any methods used to examine subgroups and interactions	NA
		(*c*) Explain how missing data were addressed	Page 6 Lines 165, and 179
		(*d*) If applicable, describe analytical methods taking account of sampling strategy	NA
		(*e*) Describe any sensitivity analyses	NA
**Results**			
Participants	13*	(a) Report numbers of individuals at each stage of study—e.g. numbers potentially eligible, examined for eligibility, confirmed eligible, included in the study, completing follow-up, and analyzed	Page 6 Line 198-199
		(b) Give reasons for non-participation at each stage	eFigure 1 in the Supplement
		(c) Consider use of a flow diagram	eFigure 1 in the Supplement
Descriptive data	14*	(a) Give characteristics of study participants (e.g. demographic, clinical, social) and information on exposures and potential confounders	eTable 9 in the Supplement
		(b) Indicate number of participants with missing data for each variable of interest	NA
Outcome data	15*	Report numbers of outcome events or summary measures	Page 7 Line 209 to Page 8 Line 245
Main results	16	(*a*) Give unadjusted estimates and, if applicable, confounder-adjusted estimates and their precision (e.g., 95% confidence interval). Make clear which confounders were adjusted for and why they were included	eTable 9 in the Supplement
		(*b*) Report category boundaries when continuous variables were categorized	eTable 9 in the Supplement
		(*c*) If relevant, consider translating estimates of relative risk into absolute risk for a meaningful time period	NA
Other analyses	17	Report other analyses done—e.g. analyses of subgroups and interactions, and sensitivity analyses	Table 1, Table 2, and Figure 3

**Discussion**			
Key results	18	Summarize key results with reference to study objectives	Page 9 Line 258 to Page 10 Line 304
Limitations	19	Discuss limitations of the study, taking into account sources of potential bias or imprecision. Discuss both direction and magnitude of any potential bias	Page 10 Line 307
Interpretation	20	Give a cautious overall interpretation of results considering objectives, limitations, multiplicity of analyses, results from similar studies, and other relevant evidence	Page 10 Line 300
Generalizability	21	Discuss the generalizability (external validity) of the study results	Page 10 Line 312
**Other information**			
Funding	22	Give the source of funding and the role of the funders for the present study and, if applicable, for the original study on which the present article is based	Page 10 Lines 321-324

*Give information separately for exposed and unexposed groups.

Note: An Explanation and Elaboration article discusses each checklist item and gives methodological background and published examples of transparent reporting. The STROBE checklist is best used in conjunction with this article (freely available on the Web sites of PLoS Medicine at http://www.plosmedicine.org/, Annals of Internal Medicine at http://www.annals.org/, and Epidemiology at http://www.epidem.com/). Information on the STROBE Initiative is available at www.strobe-statement.org.

Supplementary

eTable 2 Covariates, units, type, definitions, normal values, and UK Biobank field codes (if applicable).

Variable	Units	Type	Definition	Healthy levels	UK Biobank field code(s)
Age	years	Exposure	This is a derived variable based on date of birth and date of attending assessment center and refers to the age of the participant on the day they attended an Assessment Centre, truncated to whole year part.	-	34
Sex	-	Exposure	Female/Male	-	31
Carotid intima media thickness (CIMT)	μm	Outcome	Ultrasound measurement of the two innermost layers of the arterial wall, where atherosclerotic damage begins before plaque occurrence.[1] It is a marker of subclinical atherosclerosis.[2] IMT values of more than 0.9 mm may be attributed to asymptomatic vascular damage; European Society of Cardiology (ESC).[3]	≤ 0.9mm (ESC)	22672, 22675, 22678, 22681, 22671, 22674, 22677, 22680, 22670, 22673, 22676, 22679
Sleep duration	hours	Exposure	About how many hours sleep do you get in every 24 hours (including naps)? If the time you spend sleeping varies a lot, give the average time for a 24-hour day in the last 4 weeks	NA	1160
Physical activity score	NA	Exposure	The physical activity score was calculated using participants' self-reported answers to questions from the International Physical Activity Questionnaire (IPAQ)[4].	NA	864, 874, 884, 894, 904, 914,
Waist circumference	cm	Exposure	Waist circumference refers to the measurement taken around the abdomen at the level of the natural waist, typically just above the hip bones. It is used as a simple anthropometric indicator to assess central obesity and the distribution of body fat. Waist circumference measurement provides valuable information about the amount of fat stored in the abdominal region, which is associated with an increased risk of various health conditions, including cardiovascular diseases, type 2 diabetes, and metabolic syndrome.[5]	94-102 cm for M<br>80-88 cm for F	48
Hip circumference	cm	Exposure	Hip circumference refers to the measurement taken around the widest part of the hips and buttocks. This anthropometric measurement is valuable for evaluating body composition and fat distribution. Hip circumference is often used in conjunction with waist circumference to calculate the waist-to-hip ratio, which provides insights into the distribution of body fat and its potential health implications.[6]	-	49
Waist-hip ratio (WHR)	-	Exposure	Different patterns of fat distribution, as indicated by variations in waist-to-hip ratio, can be associated with varying levels of health risks, particularly in relation to cardiovascular and metabolic health.[6]	*≤ 0.5[7]	48, 49
Alcohol intake frequency	Categorical (single)	Exposure	ACE touchscreen question "About how often do you drink alcohol?" "Daily or almost daily", "Three or four times a week", "Once or twice a week", "Less often", and "Never"	-	1558
Smoking status	Categorical	Exposure	"0=Never", "1=Previous", "2=Current"		20116

Use of cholesterol medication		Exposure			
Use of blood pressure medication		Exposure			
Use of diabetes medication		Exposure			
Diet	Various	Exposure	Fruits and vegetables (servings/day)		Various. See tables above
Vitamin intake	Y/N	Exposure	Essential nutrients vital for bodily functions		
Mineral intake	Y/N	Exposure	Inorganic substances required for a range of physiological processes		
Townsend	0-5	Exposure	Deprivation index measuring material deprivation in a population	-	189
BMI		Exposure	Weight / $(height)^2$	18.5 – 24.9	21001
Blood pressure	mmHg	Exposure	Force exerted by circulating blood on the walls of blood vessels	90/60 mmHg and 120/80 mmHg	4079, 4080
Sedentary time	hours	Exposure	Duration of inactivity or minimal physical movement: Calculated using time spent watching TV, using computer, and driving.		1070, 1080, 1090
Physical activity	Categorical		Any bodily movement produced by skeletal muscles requiring energy expenditure.<br>Score of 2 means: high level of physical activity<br>Score of 1 means: moderate level of physical activity<br>Score of 0 means: low level of physical activity		See way above
Body weight	kg	Exposure	Total mass of a person, often used as an indicator of health		21002
WBC	x 10^9 cells/L	Exposure	White Blood Cells, a key component of the body's immune system	4 to 11 x 10^9 cells/L	30000
CRP	mg/L	Exposure	C-reactive Protein, a marker of inflammation in the body	<10 mg/L	30710
IGF-1	nmol/L	Exposure	Insulin-like Growth Factor 1, a hormone important for growth and development	1.3 – 195	30770
Cholesterol	mmol/L	Exposure	A waxy substance found in blood, essential for building cells but high levels can lead to health issues.	<5 mmol/L	30690
Triglycerides	mmol/L	Exposure	A type of fat found in the blood, used for energy or stored in the body.	<1.7 mmol/L	30870
HDL	mmol/L	Exposure	High-Density Lipoprotein, often referred to as 'good' cholesterol, which helps remove other forms of cholesterol from the bloodstream	>1mmol/L for M, >1.2mmol/L for F	30760
HbA1c	mmol/mol	Exposure	Hemoglobin A1c, a measure of average blood glucose levels over the past 2 to 3 months	20-42 mmol/mol	30750
TC:HDL ratio	-	Exposure	The ratio of total cholesterol to high-density lipoprotein, used as an indicator of heart disease risk	Below 6	30690, 30760
Medication females			Medication females (cholesterol, blood pressure, diabetes, or take exogenous hormones)		6153
Medication males			Medication males (cholesterol, blood pressure or diabetes)		6177
LDL					30780

*For Men: A WHR of 0.9 or less is considered low risk, 0.9 to 0.99 is moderate risk, and 1.0 or higher is high risk.
For Women: A WHR of 0.8 or less is considered low risk, 0.81 to 0.84 is moderate risk, and 0.85 or higher is high risk.
A normal CIMT is generally less than 0.9 mm, 0.9 mm to 1.0 mm may indicate a moderate risk of cardiovascular disease, and greater than 1.0 mm is often considered a sign of increased risk.

Supplementary

eTable 3 Food groups (used for diet score creation).

Food groups	Consisting of	Unit/frequency of intake
**Vegetable group**	Cooked vegetables† + Salad/raw vegetables	heaped tablespoons/day (1 tablespoon = 14.175 grams)
**Fruit group**	Fresh fruit + Dried fruit	pieces/day
**Unprocessed red meat group**	Beef + lamb/mutton + pork intake	0 Never<br>1 Less than once a week<br>2 Once a week<br>3 2-4 times a week<br>4 5-6 times a week<br>5 Once or more daily
**Fish group**	Oily + non-oily intake scores	0 Never<br>1 Less than once a week<br>2 Once a week<br>3 2-4 times a week<br>4 5-6 times a week<br>5 Once or more daily
**Processed meat group**	ACE touchscreen question "How often do you eat processed meats (such as bacon, ham, sausages, meat pies, kebabs, burgers, chicken nuggets)?"	0 Never<br>1 Less than once a week<br>2 Once a week<br>3 2-4 times a week<br>4 5-6 times a week<br>5 Once or more daily

†Excluding potatoes

Supplementary

eTable 4 Diet score calculations based on food groups from FFQ.

**Diet score calculations based on food groups as shown here:**	
**Fruits**	≥ 3 servings/day (A standard serve is about 150g, 350kJ)
**Vegetables**	≥ 3 servings/day (A standard serve is about 75g, 100–350kJ)
**Fish**	≥ 2 servings/week, including once a week of oily fish (A standard serve is 500–600kJ, 100g cooked fish fillet, about 115g raw, or one small can of fish)
**Processed meats**	< 1 serving/week
**Red meat (lamb/beef/pork)**	< 2 servings/week (A standard serve is 500–600kJ, 65g cooked red meats such as beef, lamb/mutton, pork (about 90-100g raw))

In developing the food groups for diet score calculations, we used specific criteria based on the UK Biobank's Food Frequency Questionnaire (FFQ) reporting methodology. Each food group was meticulously defined to ensure comprehensive dietary assessment.
The diet score calculations were based on the following food groups:

- For fruits, the criteria stipulated a consumption of three or more servings per day, with a standard serving being approximately 150 grams, equivalent to 350 kilojoules. Similarly, the vegetable group required an intake of three or more servings per day, where each standard serving was about 75 grams, providing 100 to 350 kilojoules. The vegetable group consisted of both cooked vegetables and raw salad vegetables, measured in heaped tablespoons per day, with each tablespoon equating to approximately 14.175 grams.
- The fish group was categorized based on the consumption of two or more servings per week, including at least one serving of oily fish. A standard serving for fish was defined as 100 grams of cooked fish fillet, approximately 115 grams when raw, or one small can of fish, contributing 500 to 600 kilojoules. Intake frequencies for fish were scored from 0 (never) to 5 (once or more daily).
- Processed meat consumption was limited to less than one serving per week, with intake frequencies also scored from 0 (never) to 5 (once or more daily) based on the response to the ACE touchscreen question regarding the consumption of processed meats, such as bacon, ham, sausages, meat pies, kebabs, burgers, and chicken nuggets.
- For unprocessed red meat, including lamb, beef, and pork, the criteria stipulated a consumption of less than two servings per week. Each standard serving of red meat was set at 65 grams of cooked meat, roughly equivalent to 90 to 100 grams when raw, and provided 500 to 600 kilojoules. Intake frequencies for red meat were similarly scored from 0 (never) to 5 (once or more daily).

Therefore, the diet score ranged from 1 to 4, with an individual having a score of either 1, 2, 3, or 4, corresponding to poor, fair, healthy, or optimal, respectively.

Detailed explanation:

- Diet score 1 (**Poor**): This category indicates a diet that fails to meet any of the recommended criteria. It suggests low consumption of fruits and vegetables, inadequate fish intake, and high consumption of processed and red meats.
- Diet score 2 (**Fair**): This score reflects adherence to one of the dietary guidelines. For example, the individual might be consuming adequate amounts of fruits or vegetables, or their fish intake is sufficient, but other aspects of the diet are not aligned with the recommendations.
- Diet score 3 (**Healthy**): This score suggests adherence to two of the dietary guidelines. For instance, the individual might have a good intake of both fruits and vegetables but may not meet the criteria for fish, processed meats, or red meat consumption.
- Diet score 4 (**Optimal**): This category indicates a well-balanced diet that adheres to most or all the guidelines. It suggests a diet rich in fruits and vegetables, adequate fish consumption, and limited intake of processed and red meats.

Supplementary

eTable 5 Diet groups based on dietary consumption.

Diet groups	Consisting of	Unit/frequency of intake
Vegan	Consumption of fruits, vegetables, grains and cereals, legumes and beans, nuts, and seeds **but not dairy products, eggs, fish, meat, poultry, and spreads**	Consumed/not consumed
Vegetarians	Consumption of dairy products and eggs but not fish, poultry, or red meat, i.e. lacto-ovo vegetarian;	Consumed/not consumed
Fish eaters	Consumption of dairy products, eggs, and fish but not poultry or red meat	Consumed/not consumed
White meat<br>(Fish + poultry)	Consumption of dairy products, eggs, fish, and poultry **but not red meat**	Consumed/not consumed
Meat eaters	Consumption of dairy products, eggs, fish, poultry, and red meat	Consumed/not consumed

Supplementary

eTable 6 Cardiometabolic-risk biomarker index (CRBI).

Cardiometabolic biomarker index	Optimal (0)	Intermediate (1)	Poor (2)
**HbA_{1c}**	<5.7% AND not taking diabetes medication	5.7% to 6.4% OR <5.7% AND taking diabetes medication	>6.4%
**TC:HDLr**	<3.5 AND not taking lipid lowering medication	3.5 to 5 OR <3.5 AND taking lipid lowering medication.	>5
**Blood pressure**	SBP <120 AND DBP <80 AND not taking BP-lowering medication	SBP 120-139 OR DBP 80-89 OR SBP <120 AND DBP <80 AND taking BP-lowering medication	SBP ≥140 OR DBP ≥90

Supplementary

eTable 7 Cardiometabolic-risk biomarker index (CRBI) score.

	Optimal	Intermediate	Poor
HbA1c	0	1	2
TC:HDLr	0	1	2
DBP	0	0.5	1
SBP	0	0.5	1

eTable 8 ICD-10 codes for outcomes and UK Biobank data fields.

		ICD-10 codes	UK Biobank data fields for: Date first reported	Source of report
Cardiovascular diseases	**Coronary Heart Disease**	I25	131306	131307
	**Myocardial Infarction***	I21, I22, I23	131298, 131300, 131302	131299, 131301, 131303
	**Heart Failure**	I50	131354	131355
	**Aortic Aneurysm**	I71	131382	131383
	**Peripheral Vascular Disease**	I73	131386	131387
	**Stroke (Ischemic)**	I63	42008	42009
Dementia	**Alzheimer's disease**	G30	131036	131037
	**Dementia (all cause)**	A81.0, F00, F01, F02, F03, F05, G30, G31.0, G31.1, G31.8, and I67.3	42018	42019

*Non-fatal; Date first reported = Date for diagnosis; Source of report = Source of diagnosis
Coronary Heart Disease (Acute Coronary Syndrome - ACS): ICD-10 code: I25.1 (Chronic ischemic heart disease, which includes atherosclerotic cardiovascular disease, atherosclerotic heart disease, and coronary (artery) atherosclerosis).[8]
Myocardial Infarction (MI): I21, I22, I23: ICD-10 code: I21 (Acute myocardial infarction).[8,9]
Heart Failure: ICD-10 code: (I50).[10]
Aortic Aneurysm: ICD-10 code: I71 (Aortic aneurysm and dissection)[11]
Peripheral Vascular Disease: ICD-10 code: I73.[12]
Stroke (Ischemic): ICD-10 code: I63 (Cerebral infarction)[8,9]
Dementia: ICD-10 code: F03 (Unspecified dementia) or more specific codes depending on type; F00 (Dementia in Alzheimer's disease), F01 (Vascular dementia), F02 (Dementia in other diseases classified elsewhere), F03 (Unspecified dementia), F05 (delirium superimposed on dementia) or G30 (Alzheimer's disease), G31 (Circumscribed brain atrophy), G31.1 (Senile degeneration of brain), I67.3 (Progressive vascular leukoencephalopathy).[9,13]

Supplementary

eTable 9 Participant descriptive characteristics by cIMT quartiles, (N=29292).

	Baseline characteristics*	Overall	470-600 µm	>600-670 µm	>670-750 µm	>750 µm	P-value
Anthropometrics	Total population	29292	7323	7323	7323	7323	
	Age (in years)	63.99 (7.77)	59.47 (7.02)	62.85 (7.29)	65.63 (7.22)	68.02 (6.81)	<0.001
	Female N (%)	14572 (48)					
	Ethnicity N (%)						
	White	27420 (93.84)	6791 (92.90)	6855 (93.80)	6889 (94.40)	6885 (94.30)	0.001
	Body weight (kg)	76.28 (15.00)	73.94 (14.68)	75.31 (14.82)	76.46 (14.97)	79.43 (14.98)	<0.001
	BMI	26.46 (4.31)	25.99 (4.35)	26.33 (4.35)	26.56 (4.29)	26.98 (4.21)	<0.001
	BMI$^{\#}$ N (%)						<0.001
	Underweight	211 (0.74)	68 (1.00)	67 (0.90)	52 (0.70)	24 (0.30)	
	Healthy weight	11388 (39.98)	3253 (45.60)	3001 (42.00)	2751 (38.80)	2383 (33.50)	
	Overweight	11834 (41.54)	2737 (38.40)	2843 (39.70)	2991 (42.20)	3263 (45.90)	
	Obese	5053 (17.74)	1077 (15.10)	1242 (17.40)	1297 (18.30)	1437 (20.20)	
	Waist circumference (cm)	88.39 (12.53)	85.99 (12.28)	87.35 (12.49)	88.78 (12.51)	91.46 (12.17)	<0.001
	Townsend deprivation index	-1.86 (2.73)					
Major cardiovascular risk factors	Total cholesterol (mmol/L)	5.71 (1.07)	5.58 (1.04)	5.69 (1.06)	5.75 (1.06)	5.82 (1.12)	<0.001
	LDL (mmol/L))	3.57 (0.82)	3.46 (0.80)	3.54 (0.81)	3.60 (0.81)	3.69 (0.85)	<0.001
	HDL (mmol/L)	1.47 (0.37)	1.50 (0.37)	1.50 (0.38)	1.48 (0.38)	1.41 (0.36)	<0.001
	Mean TC/HDL ratio	4.07 (1.09)					
	Triglycerides (mmol/L)	1.65 (0.96)	1.53 (0.93)	1.59 (0.93)	1.66 (0.93)	1.81 (1.02)	<0.001
	Blood pressure						
	SBP	138.49 (18.67)	131.01 (16.44)	136.44 (17.79)	140.67 (18.01)	145.89 (19.07)	<0.001
	DBP	79.05 (10.04)	78.31 (9.72)	79.11 (10.02)	79.33 (10.09)	79.44 (10.27)	<0.001
	HbA1c (mmol/mol)	34.90 (5.02)	34.06 (4.44)	34.70 (4.80)	35.11 (4.87)	35.72 (5.74)	<0.001
	C-reactive protein (mg/L)	2.04 (3.56)	1.93 (3.68)	1.98 (3.52)	2.08 (3.47)	2.15 (3.57)	0.001
	CRBI score categories N (%)						
	Optimal	2253 (7.69)	1013 (13.83)	658 (8.99)	387 (5.28)	195 (2.66)	
	Low	5661 (19.33)	1885 (25.74)	1590 (21.71)	1349 (18.42)	837 (11.43)	
	Moderate	8699 (29.70)	2173 (29.67)	2221 (30.33)	2290 (31.27)	2015 (27.52)	
	High	8416 (28.73)	1587 (21.67)	1946 (26.57)	2229 (30.44)	2654 (36.24)	
	Very high	4263 (14.55)	665 (9.08)	908 (12.40)	1068 (14.58)	1622 (22.15)	
Lifestyle risk factors	Smoking status N (%)						<0.001
	Never	18166 (62.20)	4934 (67.50)	4679 (64.00)	4488 (61.50)	4065 (55.70)	
	Former	1010 (3.50)	279 (3.80	259 (3.50)	214 (2.90)	258 (3.50)	
	Current	10041 (34.40)	2099 (28.70)	2373 (32.50)	2599 (35.60)	2970 (40.70)	
	Diet score N (%) in quarltiles						<0.001
	Optimal	3284 (11.20)	765 (10.50)	824 (11.30)	829 (11.30)	866 (11.90)	
	Healthy	10434 (35.70)	2539 (34.80)	2561 (35.10)	2666 (36.50)	2668 (36.60)	
	Fair	10712 (36.70)	2695 (36.90)	2754 (37.70)	2621 (35.90)	2642 (36.20)	
	Poor	4780 (16.40)	1306 (17.90)	1161 (15.90)	1192 (16.30)	1121 (15.40)	
	Alcohol intake N (%)						
	Never	1849 (6.30)	474 (6.50)	462 (6.30)	461 (6.30)	452 (6.20)	
	Daily or almost daily	5054 (17.30)	1082 (14.80)	1228 (16.80)	1286 (17.60)	1458 (19.90)	
	3 or 4 times a week	8374 (28.60)	2034 (27.80)	2079 (28.40)	2130 (29.10)	2131 (29.10)	
	1 or 2 a week	7761 (26.50)	2048 (28.00)	1968 (26.90)	1936 (26.40)	1809 (24.70)	
	Less often	6254 (21.40)	1685 (23.00)	1586 (21.70)	1510 (20.60)	1473 (20.10)	
	Self-reported physical activity N (%)						<0.001
	Low	5393 (18.40)	1408 (19.20)	1393 (19.00)	1342 (18.30)	1250 (17.10)	
	Moderate	12403 (42.30)	3153 (43.10)	3113 (42.50)	3078 (42.00)	3059 (41.80)	

Supplementary

Baseline characteristics*	Overall	470-600 µm	>600-670 µm	>670-750 µm	>750 µm	P-value
High	11496 (39.20)	2762 (37.70)	2817 (38.50)	2903 (39.60)	3014 (41.20)	
Sleep category (%)						0.003
<5-7hr	7015 (23.90)	1826 (24.90)	1788 (24.40)	1744 (23.80)	1657 (22.60)	
7-9hr	21912 (74.80)	5427 (74.10)	5442 (74.30)	5488 (74.90)	5555 (75.90)	
>9hr	365 (1.20)	70 (1.00)	93 (1.30)	91 (1.20)	111 (1.50)	

* Study population data is from baseline (2006-2010), cIMT values are from Imaging visit 1 (2014+). Values are Mean (SD) unless otherwise stated.
[#]BMI: Underweight (<18.50), Healthy weight (18.50 to 25.00), Overweight (25.00 to 30.00), Obese (>30.00).
CRBI score, cardiometabolic biomarker index score; IPAQ, International Physical Activity Questionnaire.
CRBI scores of 0, >0 & ≤1, >1 & ≤2, >2 & ≤3, >3 correspond to optimal, low, moderate, high, and very high (detailed categorization in eTables 6-7).

eTable 10 Multicollinearity for linear regression between CMIT and all factors.

	GVIF	Df	GVIF^(1/(2*Df))
**Age**	1.153711	1	1.074109558
**Sex**	1.456256	1	1.206754414
**Ethnicity**	1.038024	1	1.018834603
**C-reactive protein**	1.036352	1	1.018013854
**Townsend**	1.044192	1	1.021857061
**Sleep category**	1.013347	2	1.003320078
**IPAQ**	1.030149	2	1.007453411
**Diet score**	1.047552	3	1.007772646
**CRBI score**	1.304812	1	1.142283815
**Smoke score**	1.070884	2	1.017268484
**Alcohol intake**	1.104901	4	1.012547579
**Body weight**	1.487498	1	1.219630372

Supplementary

eTable 11 Association of CRBI score and individual biomarkers with cIMT.

	CRBI score		Blood pressure		TC:HDL ratio		HbA1c	
**Levels**	β (95% CI)	P-value	β (95% CI)	P-value	β (95% CI)	P-value	β (95% CI)	P-value
**Optimal**	-	-	-	-	-	-	-	-
**Low**	10.37 (5.00, 15.74)	<0.001	0.73 (-5.26, 6.72)	0.811	8.186 (5.34, 11.03)	<0.001	4.91 (1.17, 8.65)	0.010
**Moderate**	19.89 (14.63, 25.15)	<0.001	6.52 (0.46, 12.58)	0.035	16.68 (13.09, 20.28)	<0.001	9.92 (-3.04, 22.88)	0.134
**High**	32.47 (26.98, 37.97)	<0.001	20.18 (15.25, 25.15)	<0.001	28.02 (22.96, 33.07)	<0.001	11.23 (-4.03. 26.09)	0.151
**Very high**	44.38 (38.25, 50.51)	<0.001	37.50 (32.94, 42.06)	<0.001	34.53 (26.99, 42.07)	<0.001	12.85 (-5.52. 31.23)	0.170

Analyses adjusted for age, sex, ethnicity, C-reactive protein, Townsend deprivation index, sleep duration, physical activity derived from IPAQ, dietary consumption, CIMT, smoking, alcohol consumption, and body weight.

Supplementary

eTable 12 Association between cIMT and covariates, (N=29292).

		Adjusted model	
		**β (95% CI)**	**P-value**
**Anthropometrics**	**Age (in years)**	5.92 (5.74, 6.09)	<0.001
	**Sex**		
	Females	ref	
	Males	12.67 (9.63, 15.70)	<0.001
	**Ethnicity**		
	White	-7.61 (-12.96, -2.27)	<0.01
	**Weight (kg)**	0.84 (0.74, 0.94)	<0.001
	**Townsend**	-0.33 (-0.80, 0.14)	0.170
	**CRP (Inflammation)**	-0.21 (-0.57, 0.15)	0.245
	**CRBI score categories**		
	Optimal	ref	
	Low risk	10.37 (4.99, 15.74)	<0.001
	Moderate risk	19.89 (14.63, 25.15)	<0.001
	High risk	32.47 (26.68, 37.96)	<0.001
	Very high risk	44.38 (38.25, 50.51)	<0.001
**Lifestyle risk factors**	**Smoking status (%)**		
	Never	ref	
	Previous	10.16 (7.44, 12.89)	<0.001
	Current	14.71 (7.74, 21.70)	<0.001
	**Diet score (%)**		
	Poor	ref	
	Fair	1.82 (-1.91, 5.56)	0.338
	Healthy	0.54 (-3.24, 4.33)	0.780
	Optimal	1.90 (-3.02, 6.82)	0.449
	**Alcohol intake (%)**		
	Never	ref	
	~Daily	3.25 (-2.68, 9.17)	0.283
	Less often	1.20 (-4.49, 6.90)	0.677
	1-2/week	5.00 (-0.60, 10.59)	0.080
	3-4/week	3.68(-1.91, 9.26)	0.197
	**Self-reported physical activity (%)**		
	Low	ref	
	Moderate	5.11 (1.60, 8.62)	<0.01
	High	12.01 (8.43, 15.59)	<0.001
	**Sleep category (%)**		
	<7hr	ref	
	7-9hr	0.68 (-2.28, 3.63)	0.653
	>9hr	6.23 (-5.35, 17.81)	0.292

CRP, C-reactive protein (mg/L); CRBI score, cardiometabolic risk biomarker index score; IPAQ, International Physical Activity Questionnaire.
CRBI scores of 0, >0 & ≤1, >1 & ≤2, >2 & ≤3, >3 correspond to optimal, low risk, moderate risk, high risk, and very high risk.
Analyses adjusted for age, sex, ethnicity, C-reactive protein, Townsend deprivation index, sleep duration, physical activity derived from IPAQ, dietary consumption, CIMT, smoking, alcohol consumption, and body weight.

Supplementary

eTable 13 Results from the adjusted model stratified by sex. (N=29,292).

		Female		Male	
		β (95% CI)	P-value	β (95% CI)	P-value
Anthropo-metrics	**Age**	5.90 (5.67, 6.12)	<0.001	5.90 (5.67, 6.17)	<0.001
	**Ethnicity**	-6.12 (-12.48, 0.26)	0.006	-9.72 (-18.55, -0.89)	<0.05
	**Weight**	0.74 (0.61, 0.87)	<0.001	0.95 (0.80, 1.11)	<0.001
	**Townsend**	0.35 (-0.24, 0.95)	0.245	-0.94 (-1.68, -0.20)	<0.05
CVD risk factors	**CRP (Inflammation)**	-0.62 (-1.06, -0.18)	<0.01	0.36 (-0.22, 0.93)	0.228
	**CRBI score categories**	-	-	-	-
	Optimal	ref		ref	
	Low risk	26.19 (18.94, 33.44)	<0.001	5.68 (-9.45, 20.81)	0.462
	Moderate risk	52.75 (40.63, 64.88)	<0.001	17.03 (2.55, 31.51)	<0.05
	High risk	83.81 (65.68, 101.93)	<0.001	29.17 (14.71, 43.63)	<0.001
	Very high risk	111.44 (85.89, 137.00)	<0.001	42.86 (28.05, 57.67)	<0.001
Lifestyle risk factors	**Smoking**	-	-	-	-
	Never	ref		ref	
	Previous	6.95 (3.47, 10.44)	<0.001	12.96 (8.78, 17.13)	<0.001
	Current	3.09 (-6.45, 12.62)	0.526	23.32 (13.22, 33.41)	<0.001
	**Diet score**	-	-	-	-
	Poor	ref		ref	
	Fair	1.59 (-3.46, 6.64)	0.537	1.88 (-3.56, 7.32)	0.499
	Healthy	0.10 (-4.92, 5.13)	0.968	1.03 (-4.59, 6.66)	0.719
	Optimal	1.60 (-4.61, 7.81)	0.613	2.61 (-5.12, 10.35)	0.508
	**Alcohol intake**	-	-	-	-
	Never	ref		ref	
	~Daily	0.11 (-7.24, 7.46)	0.977	4.58 (-5.01, 14.08)	0.351
	Less often	1.76 (-4.85, 8.37)	0.601	0.26 (-9.48, 10.00)	0.958
	1-2/week	6.45 (-0.17, 13.07)	0.056	2.77 (-6.57, 12.11)	0.560
	3-4/week	2.55 (-4.15, 9.26)	0.455	3.69 (-5.51, 12.90)	0.432
	**Self-reported physical activity**	-	-	-	-
	Low	ref		ref	
	Moderate	0.83 (-3.62, 5.27)	0.715	8.85 (3.44, 14.27)	<0.01
	High	3.26 (-1.32, 7.84)	0.163	20.01 (14.55, 25.47)	<0.001
	**Sleep**	-	-	-	-
	<7hr	ref		ref	
	7-9hr	1.83 (-1.81, 5.47)	0.324	-0.87 (-5.55, 3.82)	0.717
	>9hr	-6.99 (-22.24, 8.26)	0.369	16.28 (-0.97, 33.54)	0.064

CRP, C-reactive protein (mg/L); CRBI score, cardiometabolic risk biomarker index score; IPAQ, International Physical Activity Questionnaire.
CRBI scores of 0, >0 & ≤1, >1 & ≤2, >2 & ≤3, >3 correspond to optimal, low risk, moderate risk, high risk, and very high risk.
Analyses adjusted for age, ethnicity, C-reactive protein, Townsend deprivation index, sleep duration, physical activity derived from IPAQ, dietary consumption, CIMT, smoking, alcohol consumption, and body weight.

Supplementary

Supplementary references

1. Newman J. Intima-media thickness (IMT). In: Gellman MD, ed. *Encyclopedia of Behavioral Medicine*. Springer International Publishing; 2020:1243-1243.
2. O'Leary DH, Polak JF, Kronmal RA, Manolio TA, Burke GL, Wolfson SK. Carotid-artery intima and media thickness as a risk factor for myocardial infarction and stroke in older adults. *New England Journal of Medicine*. 1999/01/07 1999;340(1):14-22. doi:10.1056/NEJM199901073400103
3. Iana Samova. Intima-media thickness: Appropriate evaluation and proper measurement, described. *E-Journal of the ESC Council for Cardiology Practice*. 2015;13(1)
4. Craig CL, Marshall AL, Sjöström M, et al. International physical activity questionnaire: 12-country reliability and validity. *Medicine & Science in Sports & Exercise*. 2003;35(8)doi:10.1249/01.MSS.0000078924.61453.FB
5. Ness-Abramof R, Apovian CM. Waist circumference measurement in clinical practice. *Nutrition in Clinical Practice*. 2008/08/01 2008;23(4):397-404. doi:10.1177/0884533608321700
6. Hu G, Tuomilehto J, Silventoinen K, Sarti C, Männistö S, Jousilahti P. Body mass index, waist circumference, and waist-hip ratio on the risk of total and type-specific stroke. *Archives of internal medicine*. 2007;167(13):1420-1427. doi:10.1001/archinte.167.13.1420
7. Ren C, Zhang J, Xu Y, et al. Association between carotid intima-media thickness and index of central fat distribution in middle-aged and elderly Chinese. *Cardiovascular Diabetology*. 2014/10/30 2014;13(1):139. doi:10.1186/s12933-014-0139-2
8. Sofianopoulou E, Kaptoge SK, Afzal S, et al. Estimating dose-response relationships for vitamin D with coronary heart disease, stroke, and all-cause mortality: observational and Mendelian randomisation analyses. *The Lancet Diabetes & Endocrinology*. 2024;12(1):e2-e11. doi:10.1016/S2213-8587(23)00287-5
9. UK Biobank. Algorithmically defined outcomes (ADOs). https://biobank.ndph.ox.ac.uk/ukb/ukb/docs/alg_outcome_main.pdf
10. Monica T, Gregory N, Janet T, Greg M, Francesca ND. Valvular disease burden in the modern era of percutaneous and surgical interventions: the UK Biobank. *Open Heart*. 2022;9(2):e002039. doi:10.1136/openhrt-2022-002039
11. Jia Y, Li Y, Yu J, et al. Association between metabolic dysfunction-associated fatty liver disease and abdominal aortic aneurysm. *Nutrition, Metabolism and Cardiovascular Diseases*. 2023/11/20/ 2023;doi:https://doi.org/10.1016/j.numecd.2023.11.004
12. Kogan EV, Sciria CT, Liu CF, et al. Early stroke and mortality after percutaneous left atrial appendage occlusion in patients with atrial fibrillation. *Stroke*. 2023/04/01 2023;54(4):947-954. doi:10.1161/STROKEAHA.122.041057
13. Beydoun HA, Beydoun MA, Meirelles O, et al. Cardiovascular health, infection burden, and incident dementia in the UK Biobank. *Alzheimer's & Dementia*. 2023/10/01 2023;19(10):4475-4487. doi:https://doi.org/10.1002/alz.13405
